# Milk–Ta_2_O_5_ Hybrid Memristors with Crossbar Array Structure for Bio-Organic Neuromorphic Chip Applications

**DOI:** 10.3390/nano12172978

**Published:** 2022-08-28

**Authors:** Jin-Gi Min, Hamin Park, Won-Ju Cho

**Affiliations:** 1 Department of Electronic Materials Engineering, Kwangwoon University, Gwangun-ro 20, Nowon-gu, Seoul 01897, Korea; 2 Department of Electronic Engineering, Kwangwoon University, Gwangun-ro 20, Nowon-gu, Seoul 01897, Korea

**Keywords:** organic memristors, crossbar array structure, milk, synaptic weight modulation, neuromorphic computing system

## Abstract

In this study, a high-performance bio-organic memristor with a crossbar array structure using milk as a resistive switching layer (RSL) is proposed. To ensure compatibility with the complementary metal oxide semiconductor process of milk RSL, a high-*k* Ta_2_O_5_ layer was deposited as a capping layer; this layer enables high-density, integration-capable, photolithography processes. The fabricated crossbar array memristors contain milk–Ta_2_O_5_ hybrid membranes, and they exhibit bipolar resistance switching behavior and uniform resistance distribution across hundreds of repeated test cycles. In terms of the artificial synaptic behavior and synaptic weight changes, milk–Ta_2_O_5_ hybrid crossbar array memristors have a stable analog RESET process, and the memristors are highly responsive to presynaptic stimulation via paired-pulse facilitation excitatory post-synaptic current. Moreover, spike-timing-dependent plasticity and potentiation and depression behaviors, which closely emulate long-term plasticity and modulate synaptic weights, were evaluated. Finally, an artificial neural network was designed and trained to recognize the pattern of the Modified National Institute of Standards and Technology (MNIST) digits to evaluate the capability of the neuromorphic computing system. Consequently, a high recognition rate of over 88% was achieved. Thus, the milk–Ta_2_O_5_ hybrid crossbar array memristor is a promising electronic platform for in-memory computing systems.

## 1. Introduction

In the present information age, unstructured data are rapidly increasing with the swift growth of social media platforms and artificial intelligence (AI) technologies [1,2,3]. However, processing large amounts of unstructured data is considerably challenging given the present von Neumann architecture in which the processor and memory units are separated [4,5,6]. Owing to this problem, a revolutionary structural device that can establish a novel intelligent computing platform has been developed [7,8]. Two-terminal memristors of the metal–insulator–metal (MIM) configuration have been extensively investigated in relation to the development of such computing systems, primarily owing to their geometric simplicity, nonvolatile memory, low operational power consumption, and the capability to perform computations based on sequential analog resistive switching (RS) of insulating layers [9,10,11]. For the key to the synaptic plasticity RS layer of memristors, a variety of materials, including bio-inspired, organic, inorganic, and hybrid nanocomposites, have been investigated [12,13,14,15]. In particular, memristors based on organic materials, such as pectin, albumin, chitosan, or milk, offer a variety of advantages, including low cost, high flexibility, biocompatibility, biodegradability, non-toxicity, and solution processing [16]. Accordingly, materials that offer such benefits should be compatible with state-of-the-art electronic devices such as skin-attached and wearable devices that require high flexibility with good elastic properties [17,18]. Furthermore, bio-organic memristors must resolve existing problems such as poor endurance and inconsistent long-term retention. Among many organic materials, milk is structurally superior to many organic materials and contains many molecules of a protein called casein, which is widely utilized as a component of plastics [19,20,21].

In this study, we propose a high-performance crossbar array structure for bio-organic memristors using milk as an RS layer (RSL). For fabricating the two-terminal MIM memristor, milk RSL, a solution-based biomaterial, was spin-coated on the bottom electrode (BE). Additionally, the ability to generate fine patterns by photolithography is required for the high integration of bio-organic-material-based electronic components. To facilitate the photolithography process, a high-k Ta_2_O_5_ capping layer, which serves as a mechanical and chemical protective layer, was deposited onto the milk layer to ensure compatibility with the complementary metal oxide semiconductor (CMOS) process. Subsequently, bipolar resistive switching (BRS) operation and memristive switching characteristics of the prepared crossbar array memristors were measured. Moreover, short- and long-term plasticity, which is crucial for synaptic devices, such as paired-pulse facilitation (PPF), spike-timing-dependent plasticity (STDP), and potentiation depression behaviors, were examined. Finally, the recognition rate was investigated by learning the Modified National Institute of Standards and Technology (MNIST) digits datasets to verify the applicability of the neuromorphic system.

## 2. Experimental Method

### 2.1. Materials

The crossbar array memristors were composed of: p-type (100) Si wafers (resistivity range of 1–10 Ω·cm, LG SILTRON Inc., Gumi, Korea), Ti pellets (purity > 99.999%, TFN, Seoul, Korea), Pt pellets (purity > 99.95%, TFN, Seoul, Korea), and Seoul milk (Seoul, Korea).

### 2.2. Fabrication of Milk–Ta_2_O_5_ Hybrid Crossbar Array Memristors

To start, a standard radio corporation of America (RCA) cleaning method was employed to clean the 300 nm thick thermally oxidized p-type (100) Si wafers. To create the BE of the memristors with the MIM configuration, a 10 nm thick Ti adhesive layer and a 100 nm thick Pt layer were sequentially deposited onto the substrate using an E-beam deposition system, which was followed by photolithography and lift-off processes. Subsequently, the milk RS layer, which is the most crucial part of the device, was formed as follows. The milk was filtered using polytetrafluoroethylene (PTFE) syringe filters with 1 μm pore size (Whatman, Maidstonem, UK) to eliminate particles. The filtered solution was spin-coated onto the BE at 500 rpm for 10 s and 6000 rpm for 30 s, respectively. Thereafter, it was oven-baked at a temperature of 120 °C for 30 min, which finally resulted in a solid milk layer thickness of 120 nm. Following that step, a 20 nm thick high-*k* Ta_2_O_5_ film was deposited onto the milk layer using a radio-frequency (RF) magnetron sputtering system at an operating pressure of 4 mTorr, an RF power of 75 W, and an Ar flow rate of 20 sccm. The high-*k* Ta_2_O_5_ capping layer shields the milk layer from chemical and mechanical degradation, thereby facilitating lithography processes in bio-organic-material-based devices. In fabricating the upper electrode (TE) of the MIM memristors, a 150 nm thick Ti layer was deposited onto the milk–Ta_2_O_5_ hybrid membranes using an E-beam evaporator, followed by a lift-off process. Ultimately, the contact holes in the BE were formed via a reactive ion etching (RIE) process.

**Figure 1:**
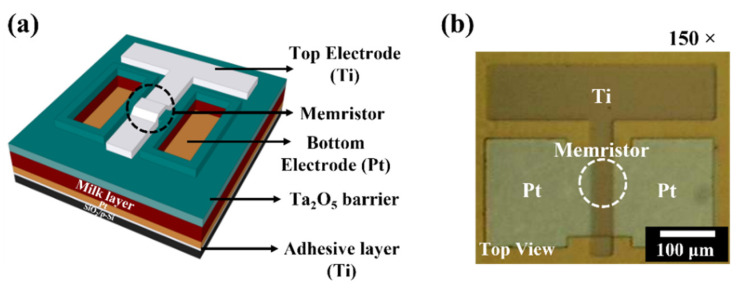
(**a**) Schematic of the fabricated milk–Ta_2_O_5_ hybrid crossbar array memristor. (**b**) Optical microscope image of the memristor (150× magnification).

Figure 1a,b illustrate the schematics of the fabricated milk–Ta_2_O_5_ hybrid crossbar array memristor and the optical microscope image of the memristor (150× magnification), respectively.

### 2.3. Characterization of Milk–Ta_2_O_5_ Hybrid Crossbar Array Memristors

The crossbar array memristors with the MIM structure were placed in a two-point probe station inside a dark box to shield from external light and electrical noise. An Agilent 4156B precision semiconductor parameter analyzer (Hewlett-Packard Co., Palo Alto, CA, USA) was used to examine the RS operation and memristive synaptic characteristics. Additionally, electrical pulse stimulation was applied to measure the synaptic modulation behavior using an Agilent 8110A pulse generator (Hewlett-Packard Co., USA). The optical microscope image of the prepared memristor was obtained using an SV-55 microscope system (SOMETECH, Seoul, Korea).

## 3. Results and Discussions

**Figure 2:**
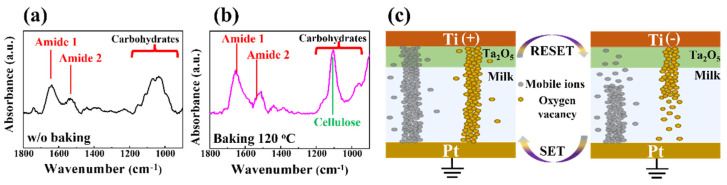
FT-IR spectra of the milk films (**a**) before and (**b**) after baking at 120 °C. (**c**) Simplified filament formation and rupture mechanism.

Before evaluating the electrical properties, it is necessary to identify the various properties of milk used as an insulating layer. Milk primarily consists of water, with 3.2% fat, 3.2% protein, 4.5% lactose, and 0.7% minerals. The composition of the milk film used in this study was investigated by analyzing the corresponding Fourier transform infrared spectroscopy (FT-IR). Figure 2a,b illustrate the FT-IR spectra of the milk films after and before baking at a temperature of 120 °C, respectively. In the FT-IR spectrum of milk, within the wavelength range of 1900–900 cm^−1^, it was found that the heterogeneous region, including vibrational bands of fatty acids, proteins, and carbohydrates, was in the range of 1700–1000 cm^−1^. The peaks at 1640 cm^−1^ and 1547 cm^−1^ correspond to protein components representing amide I (ν C = O, ν C–N) and amide II (δ N–H, ν C–N), respectively, whereas the peaks between 950 and 1200 cm^−1^ correspond to the carbohydrate (such as lactose) components (ν OH, ν C–O) [22,23,24,25,26]. Compared with the unbaked film, there was a noticeable increase in the peaks of amides I and II and carbohydrates in the film baked at 120 °C. In particular, the heat treatment altered the chain spacing and structural organization, and the 1110 cm^−1^, representing cellulose, rose rapidly [27]. It is well known that cellulose is a complex carbohydrate composed of a chain of thousands of glucose molecules. In particular, approximately 80% of the protein in milk is casein, which contains abundant mobile protons along with carbohydrates [28,29,30,31]. Figure 2c presents a simplified filament formation and rupture mechanism. The competition between the two types of conductive filaments, made of mobile ions and oxygen vacancies (V_ox_), respectively, determined the RS behaviors in the fabricated crossbar array memristors. More specifically, when a positive bias was applied to the TE, the mobile ions in the milk layer migrated to the Ta_2_O_5_ layer. In contrast, the V_ox_ in the Ta_2_O_5_ layer migrated to the milk layer. This is mainly because the components that do not form conductive filaments (CF) in each layer are diffused by the electric field owing to the relatively short distance between TE and BE.

**Figure 3:**
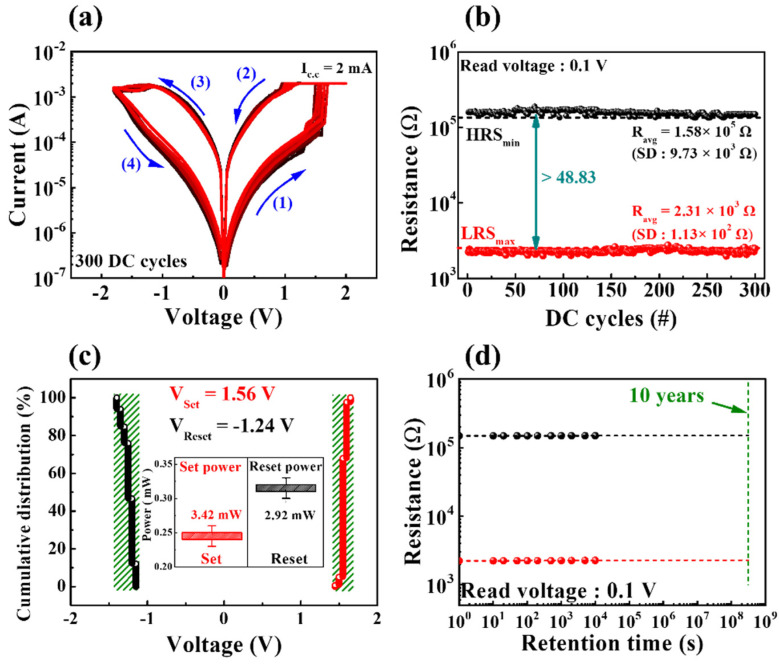
Resistive switching endurance behaviors for 300 DC cycles. (**a**) BRS *I*−*V* curves of milk-Ta_2_O_5_ hybrid crossbar array memristors. (**b**) Resistance states of milk-Ta_2_O_5_ hybrid crossbar array memristors. (**c**) Cumulative distribution of set and reset operating voltages. The calculated set and reset operating powers are displayed in the inset. (**d**) Nonvolatile retention performance of milk-Ta_2_O_5_ hybrid crossbar array memristors at a read voltage of 0.1 V.

Figure 3a depicts the endurance characteristics (for 300 DC cycles) of milk–Ta_2_O_5_ hybrid crossbar array memristors measured with a DC bias applied to TE while the BE is grounded. The figure displays that the milk–Ta_2_O_5_ hybrid crossbar array memristors provided stable BRS operation. When the BE was grounded, the current flowing through the RSL was measured by applying the consecutive DC voltage loop of 0 V → 2 V → 0 V → −1.8 V → 0 V (in 0.05 V step) to TE. When a positive voltage is applied to the TE, as indicated by arrow 1, via the SET process, the current flowing through RSL rose with the voltage. The positive electric field led the oxygen ions and mobile ions in the RSL to form the CFs. Then, the devices transitioned from the high-resistance state (HRS) to the low-resistance state (LRS) due to the CFs. Conversely, when a negative voltage was applied to the TE as indicated by arrow 4, via the RESET process, oxygen ions and mobile ions in the RSL diffused back into the CFs. The resistance state was changed from the LRS to the HRS as a result of the CFs being ruptured [32,33,34]. The high-resistance state (HRS) and the low-resistance state (LRS) were determined at a read voltage (V_read_) of 0.1 V, which was obtained from the repeated BRS I–V curves, as presented in Figure 3b. The average resistances (R_avg_) of HRS and LRS were 1.58 × 10^5^ Ω and 2.31 × 10^3^ Ω, respectively, and their corresponding standard deviations (SD) were 9.73 × 10^3^ Ω and 1.13 × 10^2^ Ω, respectively. Thus, the RS memory window, defined as minimum HRS (HRS_min_)/maximum LRS (LRS_max_), was 48.83, which was almost constant for 300 DC cycles. Figure 3c shows the cumulative distribution of the set and reset operating voltages (V_set_ and V_reset_) across 300 DC cycles. The voltage at the point where the conductance of the BRS I–V curve rapidly shifts from HRS to LRS is V_set_, whereas the voltage determined at the reset current (I_reset_) at which the conductance of the BRS I–V curve began to decline during the reset process is V_reset_ [35]. Herein, the set and reset voltages of the milk–Ta_2_O_5_ hybrid crossbar array memristor exhibited uniform cumulative distributions. The inset indicates the BRS operation power for the set process (P_set_) and reset process (P_reset_), where P_set_ = V_set_ × I_cc_ and P_reset_ = |V_reset_ × I_reset_|. The highest current in the reset process was I_reset_, whereas the compliance current (I_cc_) was a limiting current that blocks a hard breakdown during the set process. Thus, the average values of V_set_, V_reset_, P_set_, and P_reset_ were 1.56 V, –1.24 V, 3.42 mW, and 2.92 mW, respectively. Figure 3d displays the nonvolatile retention performance of LRS and HRS states at a read voltage of 0.1 V, which indicates steady nonvolatile retention properties in both resistance states.

**Figure 4:**
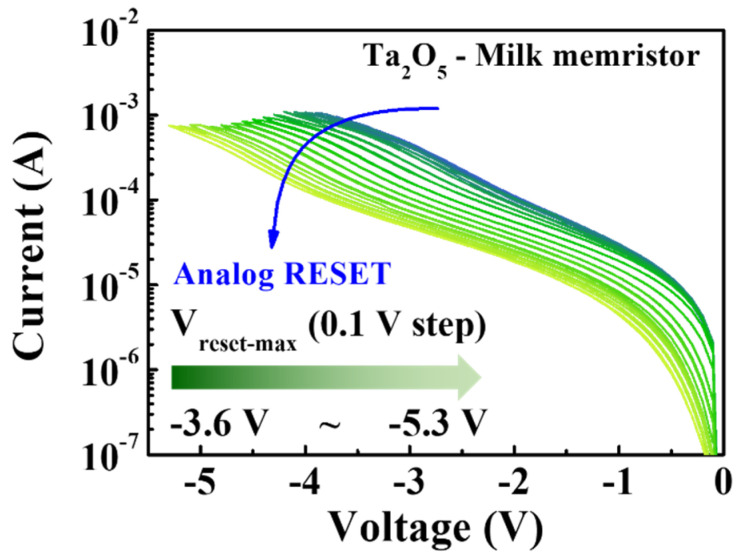
Analog RESET process of milk-Ta_2_O_5_ hybrid crossbar array memristors.

The modulation of multi-step conduction states is crucial for synaptic devices to achieve high-density memory storage. Figure 4 depicts the analog RESET process for a milk-Ta_2_O_5_ hybrid crossbar array memristor, showing how the RESET−stop voltage (V_reset-stop_) was used to regulate progressive RESET to produce multilevel memory states. The analog RESET characteristics were measured from −3.6 V to −5.3 V by consecutively reducing the maximum negative RESET voltage in steps of −0.1 V after the performance of one positive SET operation. The storage capacity corresponding to the memory window of the memristor device depends on the change in the size of I_on_/I_off_. The result derived from Figure 4 presents a method for significantly increasing the storage capacity density of a memristor device. Multilevel storage is typically accomplished by selecting the appropriate programming voltage (V_reset-stop_) or by increasing the programming current (I_cc_). However, as the conductive path is firmly established and resists breakage, a larger I_cc_ may result in a short circuit. This implies that the functional V_reset-stop_ indicates a better technique for extending I_on_/I_off_, which further leads to multilevel storage. As each of these parameters corresponds to the resistance data stored in the memory, it is challenging to change them in various HRSs.

**Figure 5:**
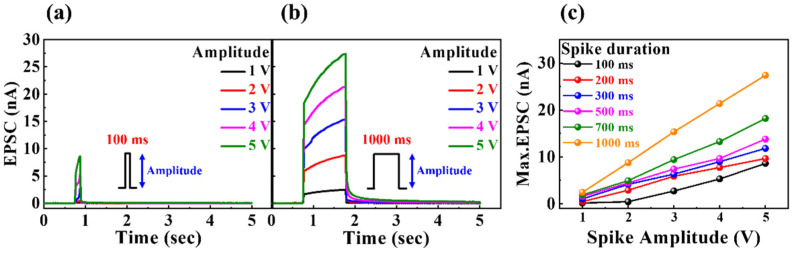
Single-spike EPSC curves of the milk–Ta_2_O_5_ hybrid crossbar array memristors with pulse duration of (**a**) 100 ms and (**b**) 1000 ms for pulse amplitudes of 1–5 V. (**c**) Maximum EPSC for various spike amplitudes and durations.

In biological nervous systems, neurons communicate with each other through synapses in response to electrical or chemical inputs. The synapses that are critical in signal transduction include the nano-clefts between pre- and post-synaptic neurons [36]. Pre-synaptic neuronal input is conveyed to the post-synaptic neuron by the diffusion of intra-synaptic neurotransmitters, the generation of a transient current, or the excitatory post-synaptic current (EPSC). Figure 5 presents the single-spike EPSC curves of milk-Ta_2_O_5_ hybrid crossbar array memristors with pulse durations of (a) 100 ms and (b) 1000 ms for pulse amplitudes of 1–5 V. Figure 5c illustrates the maximum EPSC with different spike amplitudes and durations. It is evident that smaller spike amplitudes and shorter durations result in lower EPSC values, whereas larger spike amplitudes and longer durations result in higher EPSC values. This is because additional mobile ions migrate between the electrode and the interface of the insulating layer, which results in a larger concentration gradient. Consequently, as the spike stimulus becomes stronger and longer, the ability to modulate synaptic weights for simulating human brain functions increases.

In a neurological system, PPF is crucial for processing biological temporal information, such as auditory or visual input [37]. PPF is an essential characteristic of short-term synaptic plasticity, a brain facilitation phenomenon where the first post-synaptic spike is amplified by the second pre-synaptic spike after a short time interval (Δ*t*) [38]. The second synaptic spike following the first spike induces a higher EPSC for PPF as a function of Δ*t* between two consecutive pre-synaptic spikes. The mobile ions transported by the first pre-synaptic spike diffuse between the electrolyte and the interface, where a short Δ*t* causes the mobile ions to continuously accumulate at the interface owing to the insufficient time to return to their original position [39].

**Figure 6:**
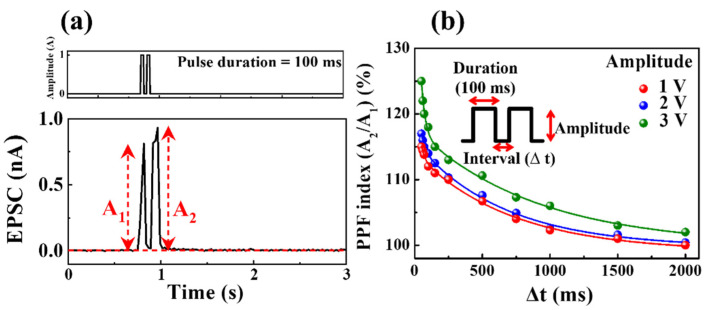
(**a**) EPSC induced by paired pre-synaptic pulses (amplitude: 1 V, duration: 100 ms, Δ*t* = 50 ms, first peak: A_1_, second peak: A_2_) on milk–Ta_2_O_5_ hybrid crossbar array memristors. (**b**) PPF index as a function of Δ*t* for two subsequent pre-synaptic pulses with amplitudes ranging from 1–3 V.

Figure 6a displays the EPSC induced by the paired pre-synaptic pulses (amplitude: 1 V, duration: 100 ms, Δ*t* = 50 ms) for milk–Ta_2_O_5_ hybrid crossbar array memristors. The second EPSC peak (A_2_) is higher than the first EPSC peak (A_1_) (A_2_ > A_1_), and partially relaxed mobile ions triggered by paired presynaptic pulses as a function of Δ*t* were responsible for the obtained PPF characteristic. Figure 6b presents a plot of the PPF index as a function of Δ*t* for two subsequent pre-synaptic pulses with amplitudes ranging from 1 to 3 V, determined by the ratio of the maximum EPSC peak amplitude (A_2_/A_1_) and Δ*t*. The PPF index varies with the length of Δt: specifically, it increases when Δ*t* is short and decreases when Δ*t* is long, thereby emulating a biological synaptic response [40]. The obtained PPF index data were fitted with the following double exponential decay relationship [38]:

(1) $$PPF\;index=A+C_{1}exp\left(-\frac{\Delta t}{\tau_{1}}\right)+C_{2}exp\left(-\frac{\Delta t}{\tau_{2}}\right),$$

where *A* is a constant, *C*_1_ and *C*_2_ represent the initial facilitation magnitudes, and *τ*_1_ and *τ*_2_ symbolize typical relaxation times. Consequently, the fitting curves of the PPF index by the double exponential decay function (solid line) were verified to be consistent with the experimental data (closed circles). Consequently, the fitting curves (solid lines) of the PPF index given by Equation (1) were verified to be consistent with the experimental data (closed circles).

**Figure 7:**
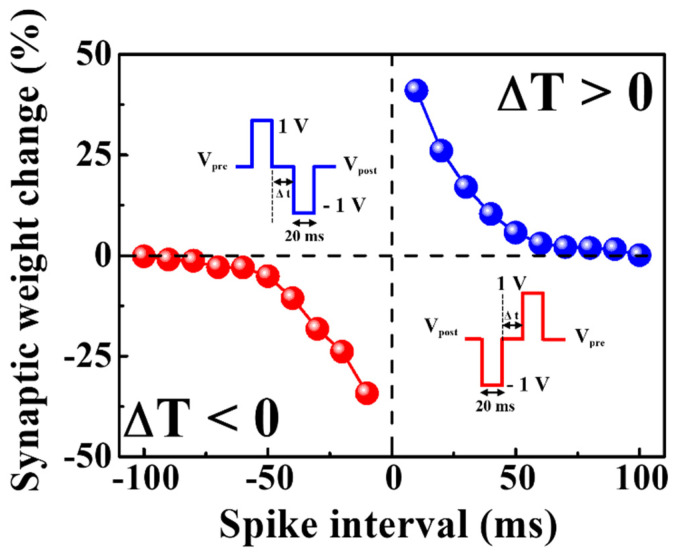
STDP properties for excitatory response modes. Illustrations of the spike signals are shown in the inset.

In contrast to short-term plasticity, long-term plasticity demonstrates long-term changes in synaptic weights and creates a template for memory storage. Rapid and repetitive stimulation strengthens the synaptic weight, and this sustained ascent is called long-term potentiation (LTP). By contrast, long-term depression (LTD) is a term used to describe a loss in synaptic weight over an extended period [41,42]. The LTP and LTD spike timings for the proposed device were identified via STDP. Pre-synaptic and post-synaptic neural learning, an important learning and memory mechanism in the brain, modulates the strength of connections between neurons by temporally correlating neural learning. STDP is an improvement on Habbian learning rules considering the temporal sequence of activities between pre- and post-synaptic neurons [43,44,45]. Figure 7 illustrates the properties of STDP for excitatory response modes. The capacity of a natural or artificial synapse to modify its strength according to the precise timing of a single pre-synaptic spike (I_1_) and a post-synaptic spike (I_2_) is referred to as STDP. The presynaptic spike arrival time (*t*_pre_) and the post-synaptic spike production time (*t*_post_) affect the synaptic weights relative to each other (Δ*T* = *t*_post_ − *t*_pre_). If the pre-synaptic spike precedes the post-synaptic spike (Δ*T* > 0), the strength of the synaptic connection increases (known as potentiation). Moreover, synaptic weights change more dramatically with shorter spike timing differences. By contrast, synaptic weights decrease when the post-spike occurs before the pre-spike (Δ*T* < 0), which indicates that the synaptic connection is inhibited (known as depression). Furthermore, as |Δ*T*| increases, the change in synaptic weight diminishes. When STDP was inverted, the milk–Ta_2_O_5_ hybrid crossbar array memristor switched to an inhibitory response mode [46]. In Figure 7, Δ*W* = (I_2_ − I_1_) produced positive values for Δ*T* > 0 and negative values for Δ*T* < 0, thus defining the relative change in the synaptic weight. The behavior of STDP is obtained by the following equation [43].

(2) $$\Delta W=\{\begin{array}{l} A^{+}exp\left(-\Delta T/\tau^{+}\right),\;\;\;\;\;\;\;\;\;\Delta T\geq 0\\ -A^{-}exp\left(\Delta T/\tau^{-}\right),\;\;\;\;\;\;\;\;\;\Delta T<0 \end{array}.$$

The range of Δ*T* is determined by *τ*^+^ and *τ*^−^, and the symbols denote the range in which synaptic connections are potentiated and depressed, respectively. The maximal synaptic alteration that can occur when Δ*t* is near to zero is determined by *A*^+^ and *A*^−^ [43,44,47,48,49]. For biological synapses, the exact pre- and post-spike timing windows that regulate the direction and magnitude of synaptic weight alterations were approximately 100ms [48,49]. Therefore, the measured data revealed that the biological STDP characteristics can be mimicked in the proposed crossbar array memristors.

**Figure 8:**
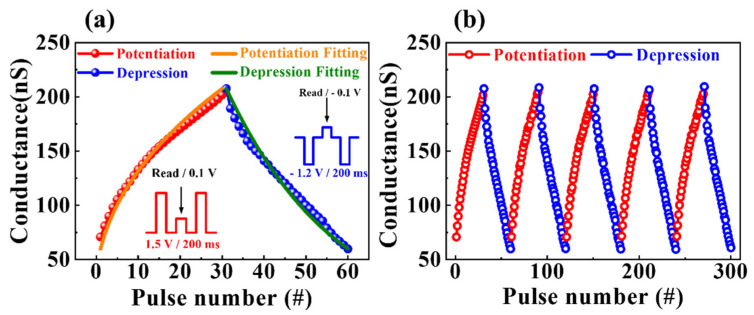
(**a**) Conductance change characteristics for 1 cycle of 30 pulses of potentiation and 30 pulses of depression. (**b**) Five cycles of successive conductance modulation operation by applying 300 pulses.

The increase and decrease in the synaptic weight features were investigated to identify the progressive conductance modulation in response to electrical pulse stimulations, which is crucial for memristive switching. Figure 8 illustrates the conductance modulation of potentiation and depression characteristics by applying pre-synaptic spikes. The conductance change characteristics for a period of 30 pulses of potentiation and 30 pulses of depression are displayed in Figure 8a. Insets represent the schematics for a single pre-spike of potentiation and depression read behavior. One cycle comprises 30 potentiation pulses (1.5 V/200 ms) and 30 depression pulses (−1.2 V/200 ms). Figure 8b shows five cycles of successive conductance modulation operation by applying 300 pulses. Over the five−cycle test period, the conductance modulation behavior with a dynamic range of approximately 150 nS was effectively modulated and remained constant. Consequently, the LTP and LTD properties of the milk-Ta_2_O_5_ hybrid crossbar array memristors induced by successive pulses were identified, and their suitability for artificial synaptic devices was demonstrated.

**Figure 9:**
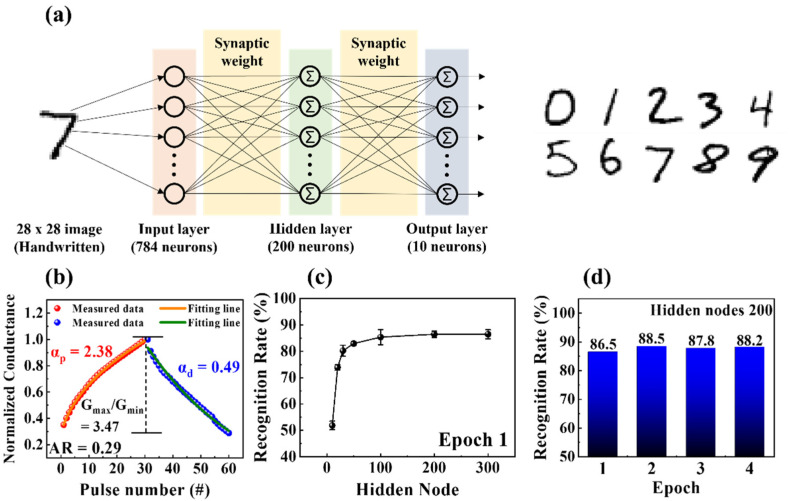
(**a**) Architecture of a three-layer fully connected ANN comprising an input layer, hidden layer, and output layer for handwritten digits recognition. The pixels of the input pattern correspond to each input neuron. (**b**) Nonlinearity analysis for normalized conductance of potentiation and depression (G/G_max_). Recognition rates according to (**c**) the number of hidden nodes at epoch 1 and (**d**) the number of epochs with 200 hidden nodes.

In biological systems, data processing, such as cognitive function pattern recognition and information transmission, is critical. A three-layer perceptron network model that simulates the learning of MNIST handwritten digits is proposed to validate that the proposed crossbar array memristors can perform neuromorphic computations. Figure 9a illustrates a designed artificial neural network (ANN) comprising an input layer (784 neurons), hidden layer (200 neurons), and output layer (10 neurons). The 28 × 28 pixels of the binarized MNIST data and the digits 0–9 were represented by 784 input neurons in the input layer and 10 output neurons in the output layer, respectively. Each neuron was connected to another via a synapse, and the synaptic weights representing the connection strength correlated with normalized potentiation and depression conductance of milk–Ta_2_O_5_ hybrid crossbar array memristors. The normalized potentiation and depression characteristics of memristors were used as the basis for MNIST pattern recognition simulations. The normalized conductance was obtained by dividing each conductance by the maximum conductance (G/G_max_), as shown in Figure 9b. The dynamic range (DR), asymmetry ratio (AR), and linearity of the normalized potentiation and depression curves play vital roles in enhancing the accuracy of learning and recognition simulations. The value of DR (G_max_/G_min_) indicates that the range of conductance modulation was 3.47. The recognition rate may change depending on the magnitude of the DR. Although high DRs do not guarantee superior performance, low DRs typically result in subpar performance [50]. The AR is an indicator of conductance modulation asymmetry. The following equation defines AR, where *G_p_(n)* and *G_d_(n)* represent the conductance values following the n_th_ potentiation or depression pulse, respectively [51]:

(3) $$AR=\frac{max\left|G_{p}\left(n\right)-G_{d}\left(n\right)\right|}{G_{p\left(30\right)}-G_{d\left(30\right)}}\;\;for\;\;n=1\;to\;30$$

For high recognition accuracy, the AR in the ideal symmetric case is preferably zero. The AR value of the milk–Ta_2_O_5_ hybrid crossbar array memristor was 0.29, which is quite close to the ideal value. Moreover, the following equation was used to derive nonlinearity factors to verify the linearity of the conductance [52].

(4) $$G=\{\begin{array}{l} {\left(\left(G_{max}^{\alpha }-G_{min}^{\alpha }\right)\times w+G_{min}^{\alpha }\right)}^{1/\alpha },\;\;if\;\alpha \neq 0\\ \;\;\;\;\;\;\;\;\;\;G_{min}\times {\left(\frac{G_{max}}{G_{min}}\right)}^{w}\;\;\;\;\;\;\;\;\;\;\;\;\;\;\;\;\;,\;\;if\;\alpha =0 \end{array},$$

where *G_max_* and *G_min_* represent the maximum and minimum conductance values, respectively. The non-linearity component controlling either the potentiation (α_p_) or depression (α_d_) is denoted by the symbol α. The internal variable *w* has a value between 0 and 1. Nonlinearity coefficient values (αp = αd = 1) are represented by changes in fully linear and symmetric conductivity in the ideal device [52,53]. The fitted potentiation and depression curves yielded nonlinearity factors of 1.33 and 0.34, respectively. Subsequently, the ANN was simulated with a dataset containing approximately 60,000 MNIST digits to calculate the recognition rate by adjusting the number of hidden nodes from 10 to 300. Figure 9c depicts the recognition rate in relation to the number of hidden nodes at epoch 1. It is evident that as the number of hidden nodes increased, the recognition rate increased until it reached a suitable level of over 86% (from 51.7%), from the starting rate of just 10 hidden nodes. Figure 9d shows the recognition rate from the first to the fourth epoch with the number of hidden nodes fixed at 200. Although the number of epochs increased, the recognition rate remained almost constant, with a high recognition rate of approximately 88 %. Thus, these outcomes demonstrate the efficiency of the proposed ANN in data processing activities, such as pattern recognition.

## 4. Conclusions

We fabricated two-terminal crossbar array memristor devices using commercial milk as an RSL. A high-k Ta_2_O_5_ capping layer on top of the milk layer facilitated the photolithography process, thereby enabling high-density integration of the device. The proposed crossbar array memristors demonstrated steady BRS characteristics, outstanding endurance, and resistance distribution for DC 300 cycle repetition. Moreover, they exhibited stable retention characteristics up to 10^4^ s. The fabricated milk–Ta_2_O_5_ hybrid crossbar array memristors featured a stable analog RESET process with memristive switching characteristics. The analog RESET process was measured by increasing the V_reset-stop_ to adjust the density of storage capacity. Additionally, the emulation of critical biological synaptic properties, such as short- and long-term plasticity, were investigated. Conductivity modulation using 300 repeated pulses (dynamic range of ~150 nS) was reliably measured by potentiation and depression. Finally, a superb recognition rate of over 88% was attained as a result of MNIST handwritten digital learning simulation by the three-layer perceptron network model. In summary, milk–Ta_2_O_5_ crossbar array memristors are expected to provide potential applications for biocompatible and environmentally friendly neuromorphic systems as a viable device for implementing artificial synapses.

## Data Availability

Not applicable.

## References

[1] Wan Q., Sharbati M.T., Erickson J.R., Du Y., Xiong F. (2019). Emerging artificial synaptic devices for neuromorphic computing. Adv. Mater. Technol..

[2] Slavakis K., Giannakis G.B., Mateos G. (2014). Modeling and optimization for big data analytics: (Statistical) learning tools for our era of data deluge. IEEE Signal Process. Mag..

[3] Pershin Y.V., Di Ventra M. (2011). Neuromorphic, digital, and quantum computation with memory circuit elements. Proc. IEEE..

[4] Indiveri G., Liu S.C. (2015). Memory and information processing in neuromorphic systems. Proc. IEEE..

[5] Wang S., Zhang D.W., Zhou P. (2019). Two-dimensional materials for synaptic electronics and neuromorphic systems. Sci. Bull..

[6] Zanotti T., Puglisi F.M., Pavan P. (2020). Smart logic-in-memory architecture for low-power non-von neumann computing. IEEE J. Electron Devices Soc..

[7] Zhang Z., Wang Z., Shi T., Bi C., Rao F., Cai Y., Zhou P. (2020). Memory materials and devices: From concept to application. InfoMat.

[8] Premsankar G., Di Francesco M., Taleb T. (2018). Edge computing for the Internet of Things: A case study. IEEE Int. Things J..

[9] Yanagida T., Nagashima K., Oka K., Kanai M., Klamchuen A., Park B.H., Kawai T. (2013). Scaling effect on unipolar and bipolar resistive switching of metal oxides. Sci. Rep..

[10] Waser R., Aono M. (2009). Nanoscience and Technology: A Collection of Reviews from Nature Journals.

[11] Khalid M. (2019). Review on various memristor models, characteristics, potential applications, and future works. Trans. Electr. Electron. Mater..

[12] Jo S.H., Chang T., Ebong I., Bhadviya B.B., Mazumder P., Lu W. (2010). Nanoscale memristor device as synapse in neuromorphic systems. Nano Lett..

[13] Ohno T., Hasegawa T., Tsuruoka T., Terabe K., Gimzewski J.K., Aono M. (2016). Short-term plasticity and long-term potentiation mimicked in single inorganic synapses. Nat. Mater..

[14] Wang Z., Joshi S., Savel’ev S.E., Jiang H., Midya R., Lin P., Hu M., Ge N., Strachan J.P., Li Z. (2017). Memristors with diffusive dynamics as synaptic emulators for neuromorphic computing. Nat. Mater..

[15] Bakan G., Gerislioglu B., Dirisaglik F., Jurado Z., Sullivan L., Dana A., Lam C., Gokirmak A., Silva H. (2016). Extracting the temperature distribution on a phase-change memory cell during crystallization. J. Appl. Phys..

[16] Raeis-Hosseini N., Lee J.S. (2017). Resistive switching memory using biomaterials. J. Electroceramics.

[17] Li Y., Qian Q., Zhu X., Li Y., Zhang M., Li J., Zhang Q. (2020). Recent advances in organic-based materials for resistive memory applications. InfoMat.

[18] Xia Y., He Y., Zhang F., Liu Y., Leng J. (2021). A review of shape memory polymers and composites: Mechanisms, materials, and applications. Adv. Mater..

[19] Seymour R.B. (1987). Nitrate (Celluloid) in 1868. Fillers were also used in casein plastic (Galalith, milk stone) in Germany in 1897 (4). Leo Baekeland used wood flour as a filler in his molded phenolic. History of Polymeric Composites.

[20] Brother G.H. (1940). Casein plastics. Ind. Eng. Chem..

[21] Jefferson M.T., Rutter C., Fraine K., Borges G.V., de Souza Santos G.M., Schoene F.A., Hurst G.A. (2020). Valorization of sour milk to form bioplastics: Friend or foe?. J. Chem. Educ..

[22] Andrade J., Pereira C.G., Almeida Junior J.C., Viana C.C.R., de Oliveira Neves L.N., da Silva P.H.F., Bell M.J.V., Anjos V.C. (2019). FTIR-ATR Determination of Protein Content to Evaluate Whey Protein Concentrate Adulteration. LWT.

[23] Lefier D., Grappin R., Pochet S. (1996). Determination of Fat, Protein, and Lactose in Raw Milk by Fourier Transform Infrared Spectroscopy and by Analysis with a Conventional Filter-Based Milk Analyzer. J. AOAC Int..

[24] Durazzo A., Gabrielli P., Manzi P. (2015). Qualitative Study of Functional Groups and Antioxidant Properties of Soy-Based Beverages Compared to Cow Milk. Antioxidants.

[25] Heuer C., Luinge H.J., Lutz E.T.G., Schukken Y.H., Van Der Maas J.H., Wilmink H., Noordhuizen J.P.T.M. (2001). Determination of Acetone in Cow Milk by Fourier Transform Infrared Spectroscopy for the Detection of Subclinical Ketosis. J. Dairy Sci..

[26] Conceição D.G., Gonçalves B.H.R., da Hora F.F.D., Faleiro A.S., Santos L.S., Ferrão S.P. (2019). Use of FTIR-ATR SpectroscopyCombined with Multivariate Analysis as a Screening Tool to Identify Adulterants in Raw Milk. J. Braz. Chem. Soc..

[27] Movasaghi Z., Rehman S., ur Rehman D.I. (2008). Fourier transform infrared (FTIR) spectroscopy of biological tissues. Appl. Spectrosc. Rev..

[28] Bresser D., Buchholz D., Moretti A., Varzi A., Passerini S. (2018). Alternative Binders for Sustainable Electrochemical Energy Storage–the Transition to Aqueous Electrode Processing and Bio-Derived Polymers. Energy Environ. Sci..

[29] Cordeschi M., Di Paola L., Marrelli L., Maschietti M. (2003). Net Proton Charge of -and -Casein in Concentrated Aqueous Electrolyte Solutions. Biophys. Chem..

[30] West I.C., Mitchell P. (1973). Stoicheiometry of Lactose–Proton Symport Across the Plasma Membrane of *Escherichia coli*. Biochem. J..

[31] Abramson J., Smirnova I., Kasho V., Verner G., Kaback H.R., Iwata S. (2003). Structure and Mechanism of the Lactose Permease of *Escherichia coli*. Science.

[32] Wong H.S.P., Lee H.Y., Yu S., Chen Y.S., Wu Y., Chen P.S., Lee B., Chen F.T., Tsai M.J. (2012). Metal–oxide RRAM. Proc. IEEE.

[33] Chen M.-C., Chang T.-C., Tsai C.-T., Huang S.-Y., Chen S.-C., Hu C.-W., Sze S.M., Tsai M.-J. (2010). Influence of electrode material on the resistive memory switching property of indium gallium zinc oxide thin films. Appl. Phys. Lett..

[34] Hsu C.H., Fan Y.S., Liu P.T. (2013). Multilevel resistive switching memory with amorphous InGaZnO-based thin film. Appl. Phys. Lett..

[35] Wu M.C., Jang W.Y., Lin C.H., Tseng T.Y. (2012). A study on low–power, nanosecond operation and multilevel bipolar resistance switching in Ti/ZrO_2_/Pt nonvolatile memory with 1T1R architecture. Semicond. Sci. Technol..

[36] Liu R., He Y., Jiang S., Wang L., Wan Q. (2021). Synaptic plasticity modulation and coincidence detection emulated in multi-terminal neuromorphic transistors. Org. Electron..

[37] Kim K., Chen C.L., Truong Q., Shen A.M., Chen Y. (2013). A carbon nanotube synapse with dynamic logic and learning. Adv. Mater..

[38] Zhou J., Liu Y., Shi Y., Wan Q. (2014). Solution-processed chitosan-gated IZO-based transistors for mimicking synaptic plasticity. IEEE Electron Device Lett..

[39] Yu F., Zhu L.Q., Xiao H., Gao W.T., Guo Y.B. (2018). Restickable oxide neuromorphic transistors with spike-timing-dependent plasticity and pavlovian associative learning activities. Adv. Funct. Mater..

[40] Majumdar S., Tan H., Qin Q.H., van Dijken S. (2019). Energy-efficient organic ferroelectric tunnel junction memristors for neuromorphic computing. Adv. Electron. Mater..

[41] Bliss T.V., Lømo T. (1973). Long-lasting potentiation of synaptic transmission in the dentate area of the anaesthetized rabbit following stimulation of the perforant path. J. Physiol..

[42] Bliss T.V., Collingridge G.L. (1993). A synaptic model of memory: Long-term potentiation in the hippocampus. Nature.

[43] Bi G.Q., Poo M.M. (1998). Synaptic modifications in cultured hippocampal neurons: Dependence on spike timing, synaptic strength, and postsynaptic cell type. J. Neurosci..

[44] Song S., Miller K.D., Abbott L.F. (2000). Competitive Hebbian learning through spike-timing-dependent synaptic plasticity. Nat. Neurosci..

[45] Feldman D.E. (2012). The spike-timing dependence of plasticity. Neuron.

[46] Sjostrom P.J., Rancz E.A., Roth A., Hausser M. (2008). Dendritic excitability and synaptic plasticity. Physiol. Rev..

[47] Kim S., Du C., Sheridan P., Ma W., Choi S., Lu W.D. (2015). Experimental demonstration of a second-order memristor and its ability to biorealistically implement synaptic plasticity. Nano Lett..

[48] Zhang L.I., Tao H.W., Holt C.E., Harris W.A., Poo M. (1998). A critical window for cooperation and competition among developing retinotectal synapses. Nature.

[49] Markram H., Lübke J., Frotscher M., Sakmann B. (1997). Regulation of synaptic efficacy by coincidence of postsynaptic APs and EPSPs. Science.

[50] Wang C., Li Y., Wang Y., Xu X., Fu M., Liu Y., Lin Z., Ling H., Gkoupidenis P., Yi M. (2021). Thin-film transistors for emerging neuromorphic electronics: Fundamentals, materials, and pattern recognition. J. Mater. Chem. C.

[51] Yang C.S., Shang D.S., Liu N., Fuller E.J., Agrawal S., Talin A.A., Li Y.Q., Shen B.G., Sun Y. (2018). All-solid-state synaptic transistor with ultralow conductance for neuromorphic computing. Adv. Funct. Mater..

[52] Jang J., Park S., Burr G.W., Hwang H., Jeong Y.H. (2015). Optimization of conductance change in Pr_1−x_ Ca_x_MnO_3_-based synaptic devices for neuromorphic systems. IEEE Electron Device Lett..

[53] Jang J., Park S., Jeong Y., Hwang H. (2014). ReRAM-based synaptic device for neuromorphic computing. 2014 IEEE International Symposium on Circuits and Systems (ISCAS).

